# Trichomonosis in Greenfinches (*Chloris chloris*) in the Netherlands 2009–2017: A Concealed Threat

**DOI:** 10.3389/fvets.2019.00425

**Published:** 2019-11-29

**Authors:** Jolianne M. Rijks, Andrea A. G. Laumen, Roy Slaterus, Julia Stahl, Andrea Gröne, Marja L. Kik

**Affiliations:** ^1^Dutch Wildlife Health Centre (DWHC), Department of Pathobiology, Faculty of Veterinary Medicine, Utrecht University, Utrecht, Netherlands; ^2^Dutch Centre for Field Ornithology (Sovon), Nijmegen, Netherlands; ^3^Pathology Division, Department of Pathobiology, Faculty of Veterinary Medicine, Utrecht University, Utrecht, Netherlands

**Keywords:** *Trichomonas gallinae*, trichomonosis, greenfinch (*Chloris chloris*), finch, bird census data, wildlife, the Netherlands

## Abstract

Finch trichomonosis in Europe is caused by a *Trichomonas gallinae* subtype A1 strain, considered to be clonal because lacking genetic heterogeneity in partial genotyping. The disease recently emerged and has been associated with a 66% reduction of the British breeding greenfinch (*Chloris chloris*) population. In contrast, in the Netherlands, where trichomonosis was detected in 2009, the breeding greenfinch population continued to grow in subsequent years. This study aimed to elucidate whether this discrepancy in population trends is because Trichomonas infection in Dutch greenfinches is associated with less severe disease, i.e., disease being less fatal. Therefore, it characterized and quantified trichomonosis in a convenience sample of greenfinches found dead and examined post-mortem between 2009 and 2017 and compared results to published data from Great Britain. Trichomonads were detected by cytology, histology, or culture in 95/101 greenfinches. The birds with trichomonads all had microscopic lesions in the upper digestive tract consistent with trichomonosis, indicating the trichomonads caused disease. The occurrence of significant lesions due to other causes was low. Some greenfinches with trichomonosis showed no macroscopic lesions. These birds showed significantly less ulceration of the mucosa and less extensive heterophil infiltration, but extent of macrophage infiltration and presence of bacteria was similar to that of birds with macroscopic lesions, and significant lesions due to other causes were equally rare. Therefore, trichomonosis was considered similarly fatal in both groups. The frequency of fatal trichomonosis in the Dutch greenfinches did not differ significantly from that reported from Great Britain. Partial genotyping of the ITS1-5,8S-ITS2 and Fe-hydrogenase regions of *T. gallinae* was performed to detect genetic heterogeneity, that could indicate the presence of other, possibly less virulent, strains. In 60/63 samples there was full alignment of sequences with the clonal strain of *T. gallinae* subtype A1. The remaining three samples had the same single synonymous nucleotide difference in the Fe-hydrogenase region; however, pathology is these three was identical to the others. Collectively, the results provide no clear evidence for less severe disease as explanation for the discrepancy in census data trends. We conclude that trichomonosis is a threat concealed in Dutch breeding greenfinch census data.

## Introduction

Trichomonosis is a potentially fatal disease of columbids (*Columbiformes*), raptors (mainly *Falconiformes*), owls (*Strigiformes*) and other selected bird species. It can cause host population declines when emerging in susceptible populations and thus present a serious threat for endangered species (1–3).

The protozoal parasite *Trichomonas gallinae* is the prime etiological agent of trichomonosis, and columbid species are considered primary hosts (4). The transfer of a single trichomonad is enough to cause infection (5). The parasite invades the mucosa of the upper digestive tract, causing damage to the epithelium and then invading mucosa and submucosa (6). There are multiple strains of *T. gallinae*, that can be classified into clades based on partial genome sequences (7). The strains vary in pathogenicity, but there is no molecular assay to distinguish virulent from non-virulent strains (1). The most virulent strains can cause death within 4 days after infection (8). Birds infected early in life by avirulent or moderately virulent strains can develop protective immunity to virulent strains, and may be able to carry virulent strains (1, 4, 8–10). Co-evolution between host and pathogen is probable, given the strong selective pressure (11).

Wild passerine species are not commonly infected by trichomonads (1, 12). However, recently a strain of *T. gallinae* subtype A1 has been infecting finches (*Fringillidae*) in Great Britain (GB) and elsewhere Europe (13–18, 20–23). This strain has unique sequences in partial genotyping (22), suggesting that finch trichomonosis is possibly caused by a single, clonal strain. The emergence of the disease in finches is associated with high mortality and population declines (13–18, 20–23). Trichomonosis accounted for 80–100% of the deaths caused by infectious disease in finches examined post-mortem in GB during 2006–2016 (14, 24). Greenfinches (*Chloris chloris*) have especially been affected (14), and the breeding greenfinch population in GB has declined steadily since the disease was detected from approximately 4.3 million in 2006 to 1.5 million in 2016 (24). This marked decline by 66% has shown no sign of leveling off. As a result of the decline, the breeding greenfinch population in GB was classified as endangered in an assessment based on the International Union for Conservation of Nature (IUCN) guidelines for extinction risk (24, 25). In contrast, in the Netherlands (the NL), where trichomonosis was first detected in greenfinches in 2009^1^, the Dutch breeding greenfinch population continues to grow. Only in wintering (including migrating) greenfinches was there a decline in numbers (Supplemental Material 1).

The cause for this discrepancy in breeding greenfinch population trends between the NL and GB in the presence of trichomonosis is unclear. Therefore, trichomonas-associated mortality in greenfinches in the NL was compared to trichomonas-associated mortality in greenfinches elsewhere, specifically in GB, to answer the question whether infection in Dutch greenfinches caused less severe disease, i.e., disease being less fatal. The aim of this study was therefore to characterize and quantify trichomonosis in a convenience sample of greenfinches found dead and examined post-mortem between 2009 and 2017 in the NL. These data were then compared to data published on *T. gallinae* subtype A1 finch trichomonosis in greenfinches in GB. The hypotheses were that in the Dutch sample trichomonosis would account significantly less frequently for greenfinch deaths than in greenfinches examined post-mortem in GB, and that the partially typed trichomonas genomes would show genetic heterogeneity.

## Materials and Methods

### Materials

Since 2009, greenfinches found dead have been submitted to the Dutch Wildlife Health Centre (DWHC) for post-mortem examination in the context of wildlife disease scanning (under permit no. FF/75A/2008/075). In 2016, submissions of greenfinches found dead were actively encouraged through media. The greenfinches were examined as soon as possible after the call from the submitter, which usually meant the next day. The birds were kept cool by the submitter and refrigerated at 4°C during transport and overnight. They were never frozen. Post-mortem examination was performed using a standard examination protocol including histology to identify lesions and probable cause of death.

### Trichomonad Status

Trichomonads were detected microscopically using one to three of the following methods: cytological examination of smears of macroscopic trichomonas-like lesions; histological examination of predilection tissues (hematoxylin-eosin staining); and trichomonas culture on gape, pharynx, esophagus and/or crop tissues. For the latter, tissues were incubated at 30°C (22) in DMEM (Gibco; Thermofisher, the NL) with 10% fetal bovine serum, supplemented with 1% penicillin/streptomycin (Gibco). The cultures were checked daily for the presence of motile trichomonads using the hanging drop technique. When 10–15 or more trichomonads were observed per image at 100X magnification, the tissue sample was removed, and the remaining cultures were centrifuged (5 min, 3,500 rpm). The supernatants were poured off and the pellets were suspended in approximately 100 μL clinging medium and stored at −80°C.

For analysis, the proportion of greenfinches infected with trichomonads was first assessed. Birds were classified into “trichomonads present,” “trichomonads absent,” and “inconclusive” specimens. “Trichomonads present” was a greenfinch with trichomonads detected by at least one test. The assumption was made that all trichomonads observed belonged to the genus *Trichomonas*. “Trichomonads absent” was a greenfinch tested by at least two methods to account for the limited negative predictive value of the tests and conclusively negative in all tests performed (12, 18). The specimens for which the trichomonad status could not be concluded according to the above definitions were considered “inconclusive” and excluded from the subsequent investigation steps. Greenfinches that were found on one day at one location were considered to belong to one mortality event.

Presence of trichomonads does not necessarily indicate disease, but it is assumed that the presence of lesions characteristic of trichomonosis and its complications, and the absence of other significant lesions, strongly support fatal trichomonosis. To be able to investigate whether greenfinches without macroscopic lesions were more likely to be cases of mild, subclinical or non-clinical disease, a distinction was made throughout the analysis between specimens with and without macroscopic lesions in the upper digestive tract.

### Microscopic Lesions Upper Digestive Tract

To assess presence and severity of microscopic lesions at trichomonad-predilection sites in infected specimens, four characteristic attributes of trichomonad infections were scored in affected tissues (pharynx, esophagus or crop): the depth of the ulceration (0 if no ulceration; 1 if erosion of the epithelium only; 2 if ulceration of epithelium and submucosa only; 3 if transmural ulceration), the extent of macrophage and heterophilic granulocyte infiltration assessed on the basis of five high power fields (0 if no or just one inflammatory cells; 1 if the inflammatory cells comprised <5% of the analyzed tissue; was seen; 1 if the inflammatory cell comprised <5% of the analyzed tissue; 2 if the inflammatory cells comprised 5–50% of the analyzed tissue; 3 if the inflammatory cells comprised >50% of the analyzed tissue), and the presence of bacteria (0 if not present; 1 if present). In addition, the visibility of trichomonads was noted (0 if not visible; 1 if visible). The slides were evaluated by two persons, including one veterinary pathologist. When there were discrepancies, the parties met to reach a consensus. One-sided Fisher exact tests (*p* = 0.05) were performed in R Core Team (19) to investigate if the scored attributes were more severe in specimens with macroscopic lesions than without.

### Contribution of Trichomonosis to Mortality

The proportions of trichomonad-infected specimens with and without other significant lesions was then determined to conclude the relative contribution of trichomonosis to greenfinch mortality in the NL. For this analysis, hemorrhagic diathesis in trichomonad-infected specimens with upper digestive tract lesions was considered a complication of trichomonosis and not a distinct lesion. The reason is that lesions in the mucosa of the upper digestive tract can result in reduced food intake and starvation, which results in hemorrhagic diathesis in passerine birds (26). Chi-square tests (*p* = 0.05) were performed in R to compare the contribution of hemorrhagic diatheses to death in trichomonad-infected specimens with and without other significant lesions, and to examine if there was a significant difference in presence of substantial lesions due to other causes between greenfinches with and without macroscopic trichomonosis lesions.

### Statistical Comparison With GB Mortality Data

The mortality data from the NL was then compared to a study on greenfinches examined post-mortem in GB from 1 April to 30 September 2006, i.e., the two seasons when most cases occur. Trichomonosis was diagnosed in 70 of 125 specimens based on macroscopic lesions (termed “necrotic ingluvitis” in the study) and *T. gallinae* detection by culture, PCR or both, and trichomonosis was considered probable in 20 of 125 specimens based on macroscopic lesions consistent with trichomonosis with negative *Salmonella* sp. culture (23). This total of 90 of 125 specimens was used for comparison to the proportion of trichomonad-infected greenfinches with macroscopic lesions in the NL.

In addition, the ratio of deaths due to trichomonosis over the total of deaths due to infectious disease was compared between GB (372/426, 2010–2016) (24) and the NL (the NL data, 2009–2017). For consistency with the criteria used in the GB study, the numerator of the NL sample consisted of the trichomonosis birds with no concurrent other infectious disease, and the denominator of all specimens with infectious disease. The Chi-square tests were performed in R.

### Trichomonad Partial Genome Sequencing

The genetic heterogeneity of the trichomonads present in the greenfinches was investigated based on the partial sequence identity of the ITS region and the Fe-hydrogenase gene. The ITS1-5,8S-ITS2 locus served to distinguish *T. gallinae* from other trichomonad species (1) and to determine the major clade, A or B (2, 7), the more variable Fe-hydrogenase gene for further differentiation among strains (2). The obtained sequences were compared to those of the epizootic “clonal” strain (22).

In brief, DNA was extracted from frozen and subsequently thawed trichomonad pellets using the QiaAmp DNA Mini Kit (Qiagen, UK) following the manufacturer's instructions. Isolated DNA was stored at 4°C for a short period (a few days) or at −80°C for a longer period (week to months). The concentration and purity of the DNA isolates were checked using the Nanodrop 1000 Spectrophotometer V3.8.1. For amplification of the ITS1-5.8S-ITS2 gene, previously established primers were used: TFR1 primer with minor modifications, as indicated in bold [5′ TGCTTCAGTTCAGCGGGT**TC**TCC 3′] and TFR2 (27). For the amplification of the Fe-dehydrogenase gene the previously established primers TrichhydFOR and TrichhydREV were used (22). PCR amplification was performed with a PCR Master Mix (Phusion). The ITS1/5,8S/ITS2 cycling parameters consisted of an initial denaturation at 98°C for 30 s, followed by 35 cycles at 98°C for 10 s and at 72°C for 30 s, and 72°C for 7 min for the final extension step. The Fe-hydrogenase cycling parameters for Fe-hydrogenase consisted initial denaturation at 98°C for 30 s, followed by 35 cycles at 98°C for 10 s, 65°C for 30 s, 72°C for 60 s, and 72°C for 7 min for the final extension step. Positive and negative controls (distilled water) were included in all PCR runs. The PCR products were purified and were sequenced by Macrogen (the NL). The obtained sequences were aligned with the SeqMan Pro program of Lasergene version 12.0 (DNAStar Inc., Madison Wisconsin) and were analyzed manually. BLAST was used for comparison. Sequences were deposited in GenBank under the following accession numbers: MN385401 (for ITS1/5,8S/ITS2 region of greenfinch no 3), MN385402, MN385403, MN385404 and MN385405 (for Fe-hydrogenase region of greenfinches no 3, 38, 84, and 94, respectively).

### Spatial Distribution and Seasonal Occurrence

Results were mapped and plotted per season for overview, against a background of reports of diseased greenfinches made to the Dutch Center for Field Ornithology (Sovon) during 2009–2017. The spatial distribution was mapped using ArcGIS software. The seasons were spring (21 March−20 June), summer (21 June−20 September), autumn (21 September−20 December) and winter (21 December−20 March).

## Results

### Trichomonad Status

In total, 123 greenfinches submitted from 95 mortality events were examined post-mortem between 1 August 2009 and 1 June 2017 (Supplemental Material 2). The presence or absence of trichomonads could be determined for 101/123 greenfinches (Table 1; Supplemental Materials 2, 3). These 101 greenfinches were found during 78 mortality events. In the remaining 22/123 specimens, the trichomonad status was “inconclusive.” This was generally due to the autolytic state of the specimen and they were excluded. Excluding the inconclusive results, the concordance between tests was 80% (Supplemental Material 3). The hanging drop following culture was more sensitive than histology and cytology (Supplemental Material 3).

**Table 1:** Significant lesions and probable cause of death of the 123 greenfinches grouped according to trichomonad presence and macroscopic lesion detection.

**Trichomonads**	**Macroscopic** **lesions upper** **digestive tract**	**Condition**	Significant lesions, probable cause of death^*^
Present (95)	Detected (56)	Cachectic (22), poor (17), moderate (2), good (2)	Trichomonosis (43): trichomonosis only (15), with HD (26); trichomonads only (1), with HD (1)
		Cachectic (4), poor (7), moderate (1)	Trichomonosis and other (13): trichomonosis and trauma (4), and campylobacter enteritis (1), and pneumonia unknown etiology (1), and pneumonia and hepatitis unknown etiology (1), with HD and trauma (3), with HD and coccidiosis (1), with HD and fungal pneumonia (1); trichomonads and salmonellosis (ingluvitis, pneumonia; 1)
	Not detected (37)	Cachectic (16), poor (10), moderate (3)	Trichomonosis (29): trichomonosis only (8), with HD (15); trichomonads only (1), with HD (5)
		Cachectic (4), poor (2), moderate (2)	Trichomonosis and other (8): trichomonosis and trauma (3), and trauma and bacterial pneumonia (1); trichomonads and atoxoplasmosis (1), and trauma (1), with HD and trauma (1), with HD and campylobacter enteritis (1)
	Not determined (2)	Not determined (2)	Not determined (2): too autolytic, but trichomonads cultured (2)
Absent (6)	Detected (1)	Poor (1)	Salmonellosis (sepsis; 1)
	Not detected (5)	Cachectic (1), poor (2), moderate (2), good (1)	Yersiniosis (sepsis; 1), trauma (3), unknown (1)
Inconclusive (22)	Detected (5)	Cachectic (4), poor (1)	Salmonellosis (sepsis; 1), avipox ingluvitis with bacterial sepsis (1), stomatitis unknown etiology (1), avian malaria (1), unknown (1)
	Not detected (14)	Cachectic (6), poor (3), moderate (2), good (2)	Salmonellosis (sepsis; 1), HD (3), pharyngitis or ingluvitis with HD (2), avian malaria with HD (1), trauma (2), cardiopathy (1), unknown (4)
	Not determined (3)	Not determined (3)	Unknown (3)

*HD, Hemorrhagic diathesis G.I. tract*.

* *“Trichomonads” indicates the specimen was infected with trichomonads but trichomonas-associated lesions at predilection sites could not be assessed histologically, generally due to autolysis. These specimens are indicated separately for completeness but viewed as trichomonosis cases in this table since in specimens where histological examination was possible, the microscopic lesions that are indicative of trichomonas-associated disease were always found, even when there were no apparent macroscopic lesions*.

Trichomonads were absent in 6/101 specimens. These six specimens were found dead in six (6/78) mortality events. Their deaths were associated with bacterial sepsis (2/6), trauma (3/6) or due to an unknown cause (1/6) (Table 1). Trichomonads were present in the remaining 95/101 greenfinches (Table 1). These 95 specimens were found dead in 72 of the 78 mortality events.

**Figure 1:**
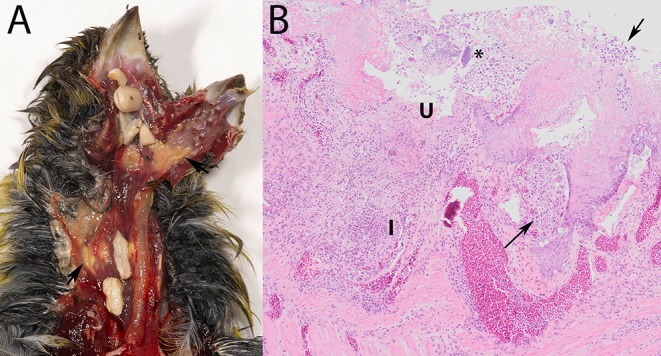
Trichomonosis in a greenfinch (*Chloris chloris*). **(A)** Macroscopic lesions, consisting of caseous yellowish material on the hyperemic mucosa of the upper digestive tract, with seeds stuck. **(B)** Lesions associated with trichomonads (arrows) in the crop, demonstrating the scored microscopic characteristics depth of ulceration (U), extent of macrophage and of heterophilic granulocyte infiltration (I) and presence of bacteria (*) (HE stain, 10x).

Macroscopic lesions, consisting of caseous yellowish material on the hyperemic mucosa of the gape, pharynx, esophagus or crop or several of these (Figure 1A), could be assessed for 93/95 specimens (Table 1), and were observed in 56/93 (60%). Seeds adhered firmly to the mucosa in 14/56 of the birds with macroscopic lesions (Figure 1A). No macroscopic lesions were present in 37/93 (40%) birds. Seeds adhered firmly to the mucosa in 4/37 specimens without visible lesions. In summary, trichomonads were present in a high proportion of the greenfinches, but these birds did not always have macroscopic lesions indicative of trichomonosis.

### Microscopic Lesions Upper Digestive Tract

Trichomonad infection in the examined greenfinches was observed only in the mucosa of the upper digestive tract. No trichomonas-associated lesions were observed elsewhere in the head (sinuses, orbital regions, brain, or neck), lung, or liver.

Characteristically, the affected mucosa was thickened due to epithelial hyperplasia and covered by a mixture consisting of fibrin or serum, cellular debris, erythrocytes and trichomonads, occasionally also with bacteria. The trichomonads were visible in most infected specimens as leaf-shaped to ovoid pale pink organisms among epithelial cells, in the submucosa and muscular layers (Figure 1B). Areas of multifocal to widespread necrosis, erosion or ulceration, interrupted the hyperplastic epithelium (Figure 1B).

Such microscopic lesions were present in all trichomonad-infected specimens, that were histologically examined (Table 2). However, the depth of ulceration was significantly less profound in cases without macroscopic lesions, i.e., those with microscopic lesions only (*p* = 0.032, Table 2). Variable degrees of submucosal and occasionally transmural inflammation, composed of heterophilic granulocytes and macrophages, lymphocytes and plasma cells were present (Figure 1B). The extent of heterophilic granulocyte infiltration was significantly less in cases without macroscopic lesions (*p* = 0.019; Table 2). In contrast, macrophage infiltration was similar between both groups (*p* = 0.313; Table 2). Bacterial colonization (Figure 1B) was also comparable in in both groups (*p* = 0.139; Table 2). In summary, all histologically examined trichomonad-infected greenfinches had microscopic necrotizing lesions in the upper digestive tract. Those without macroscopic lesions had significantly less deep ulcerative lesions and less extensive heterophil infiltration, but extent of macrophage infiltration and presence of bacteria was similar to that of specimens with macroscopic lesions.

**Table 2:** Presence and severity of microscopic lesions in the upper digestive tract of 51 greenfinches infected with trichomonads, distinguishing between specimens with and without macroscopic lesions in the upper digestive tract.

**Characteristic**	**Scoring category**	**Trichomonas infection with** **visible macroscopic** **lesions (*n* = 39)**	**Trichomonas infection without** **visible macroscopic** **lesions (*n* = 12)**	***P*-value one sided** **Fisher's exact test**
Ulceration	Absent	0	0	0.032
	Epithelium only (erosion)	3	4	
	Epithelium and submucosa	17	6	
	Transmural	19	2	
Macrophages	None or just one	9	1	0.313
	<5% of tissue	14	8	
	5–50% of tissue	7	2	
	>50% of tissue	9	1	
Heterophilic granulocytes	None or just one	4	4	0.019
	<5% of tissue	10	6	
	5–50% of tissue	6	1	
	>50% of tissue	19	1	
Bacteria	Absent	1	2	0.139
	Present	38	10	

### Contribution of Trichomonosis to Mortality

Trichomonosis was often severe enough to have caused death, with or without reaching the stage of macroscopically visible lesions. Based on post-mortem examination, 72/93 (77%) trichomonad-infected greenfinches died from trichomonosis alone or from trichomonosis with hemorrhagic diathesis (Table 1). Among these, 43/72 had macroscopic lesions in the upper digestive tract, whereas 29/72 had not. The remaining 21/93 (23%) birds had other significant lesions in addition to trichomonosis or trichomonosis with hemorrhagic diathesis. These other significant lesions were associated with bacterial (4/21), protozoal (2/21) or fungal (1/21) infections, pneumonia (2/21) or hepatitis (1/21) of unknown etiology, or trauma (13/21). Of the 21 trichomonad-infected birds that also had other significant lesions, 13/21 had macroscopic lesions in the upper digestive tract and 8/21 had not (Table 1). Hemorrhagic diathesis occurred significantly more frequently in the trichomonad-infected birds without lesions due to other causes (47/72; 65%) than in those with (7/21; 33%) (*X*^2^ = 6.813; *p* = 0.009), consistent with a contribution of this complication to fatal trichomonosis. The specimens without macroscopic lesions did not have significantly more other lesions (8/37; 21%) than specimens with macroscopic lesions (13/56; 23%) (*X*^2^ < 0.001; *p* = 1), suggesting that trichomonosis can be equally fatal in cases with or without macroscopic lesions.

### Statistical Comparison With GB Mortality Data

Statistical comparison with published GB mortality data provided no evidence for significant differences in trichomonosis disease in greenfinches between the NL and GB. The proportion of specimens in the Dutch cohort with trichomonads and macroscopic lesions (56/93, 60%) did not differ significantly from the published proportion of greenfinches with macroscopic lesions associated with trichomonosis in GB (90/125, 72%; Chi-square test *X*^2^ = 2.837; *p* = 0.092) (23). Also, the ratio of the number of trichomonosis cases with no concurrent other infectious disease (82 = 93–9) over the total number of greenfinches with infectious disease (95 = 93 + 2; Table 1) is 86% (82/95). This ratio does not differ significantly from that of GB (372/426; 87%) (*X*^2^ = 0.009; *p* = 0.924) (24).

### Trichomonad Partial Genome Sequencing

PCR products were obtained from the trichomonad pellets of 63 greenfinches belonging to 50 mortality events occurring in 2010 (1/63), 2013 (2/63), 2014 (1/63), 2015 (1/63), and 2016 (58/63) (Supplemental Material 2). The sample consisted of birds with or without macroscopic lesions of trichomonosis and with or without significant lesions due to other causes (Table 3).

**Table 3:** Overview of the partial genotyping results for *Trichomonas* according to the lesions observed in the 63 tested greenfinches.

**Trichomonosis-** **associated macroscopic** **lesions**	**Significant lesions due** **to other causes**	**ITS1/5,8/ITS2 gene** **100% sequence identity**	**Fe-hydrogenase gene** **100% sequence identity**	**Fe-hydrogenase gene 99.89%** **sequence identity (1 synonymous T-** **to-C substitution at position 135)**
Yes	No	31	29	2
Yes	Yes	10	10	
No	No	15	14	1
No	Yes	5	5	
No pathology performed	Not determined	2	2	
Total		63	60	3

*The sequences were compared to the “clonal” finch trichomonosis T. gallinae subtype A1 strain present in the GB (GenBank accession numbers GQ150752 for the ITS1/5,8/ITS2 gene and JF681136 for the Fe-hydrogenase gene)*.

All trichomonads were *T. gallinae*. All 63 PCR products (326 bp) obtained for the ITS1-5,8S-ITS2 region showed 100% sequence identity (100% query coverage) with recent GenBank accessions for *T. gallinae* in greenfinches (MK172847) (20). The nucleotide sequences also had 100% sequence identity (66% query coverage) to GenBank accession number GQ150752, the sequence obtained for the ITS1-5,8S-ITS2 region from an infected greenfinch in GB (214 bp) (23).

There was restricted evidence for genetic heterogeneity in the Fe-hydrogenase gene. The sequences of 60/63 PCR products (901 bp) obtained for the Fe-hydrogenase gene fully aligned with the sequence of the of 901 bp of the “clonal” strain (Genbank accession number JF681136). In the other three cases, all from 2016, one nucleotide difference was seen when compared to the same clonal strain (99.89% identity). In all three, there was a synonymous T-to-C substitution at position 135 compared to JF681136. Pathology in these three birds was not distinct from other trichomonas-infected specimens (Table 3; Supplemental Material 2).

### Spatial Distribution and Seasonal Occurrence

**Figure 2:**
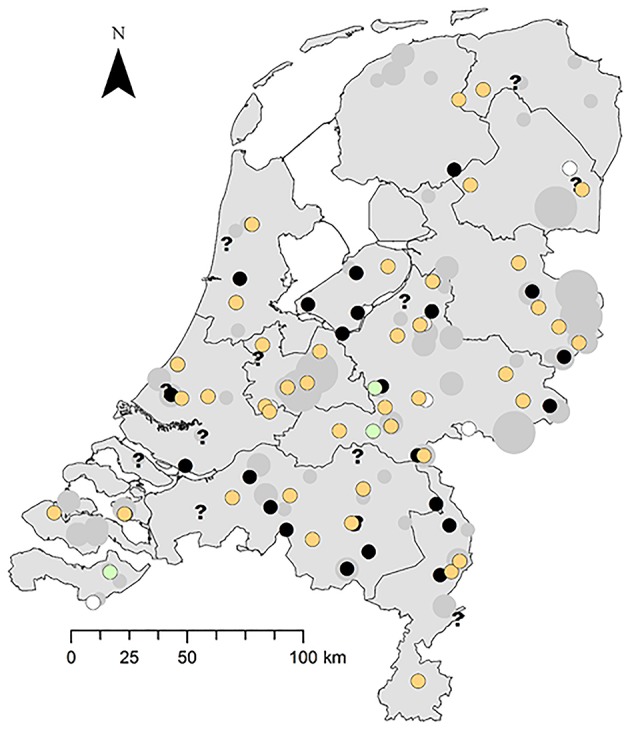
Spatial distribution of trichomonosis cases in the period 2009–2017 against the background of diseased greenfinches reported to Sovon. Trichomonad status: trichomonads subtype A1 present, orange circle; trichomonads subtype A1 with one substitution, green circle; trichomonads present, not genotyped, black circle; trichomonads absent, white circle; inconclusive, question mark. Number of diseased specimens reported to Sovon: 1, small gray dot; 2–10, middle-sized gray dot; >10 large gray dot.

The disease occurs widespread and has a seasonal peak. The greenfinches infected with trichomonosis were found widely across the NL (Figure 2). Trichomonosis cases were mostly reported during the spring and summer months; however, some cases did occur in autumn and winter (Supplemental Material 4).

## Discussion

The Dutch greenfinch breeding population continued to grow in the years following the first detection of finch trichomonosis in 2009, unlike the GB population where a decline set in directly when the disease emerged in 2005 and where the disease is perceived as a threat to greenfinches (24, 25). One of the possible explanations for this discrepancy is that the emergence of *Trichomonas* infection in Dutch greenfinches is associated with less severe disease, i.e., disease being less fatal. This possibility was investigated in this study by analyzing the contribution of trichomonosis to greenfinch mortality and the genetic heterogeneity of *Trichomonas* strains. This was done in a convenience sample of greenfinches found dead between 2009 and 2017 in the NL, and through comparison of data to those published from GB. The investigation found no evidence that trichomonosis in the Dutch sample accounted significantly less frequently for greenfinch deaths than in greenfinches examined post-mortem in GB, and only restricted evidence for genetic heterogeneity. Within the limits of partial genetic genotyping, the presence of the same strain that causes finch trichomonosis in GB was confirmed. In addition, a strain with a single synonymous substitution was detected in very few samples. However, the strain with the substitution was also associated with severe disease. Collectively, the results provide no clear evidence for less severe disease as explanation for the discrepancy in census data trends. It is concluded that trichomonosis in NL and GB is similarly fatal for greenfinches. The potential impact on the Dutch greenfinch breeding population may not (yet) be visible in the census data, but the threat is present.

To establish whether trichomonosis in the Dutch sample accounted significantly less frequently for greenfinch deaths than in greenfinches examined post-mortem in GB, it was first necessary to detect the causative agent, then to assess the contribution of the disease to death and compare the data to GB data. This study used three methods that detect trichomonads microscopically: cytological examination, histological examination and culture with hanging-drop examination. Culture with hanging-drop examination has been used as a diagnostic “gold standard” (12), and concordantly had relatively greater sensitivity for detecting trichomonads microscopically than cytology and histology. Trichomonads were present in many of the birds examined. The PCR-tests and sequencing on the cultured products confirmed that these trichomonads were *T. gallinae*, the causal agent of trichomonosis. Only a small number of birds classified as “trichomonads absent” based on “a minimum of two examination methods performed and conclusively negative by all examination methods performed.” These are rather demanding criteria for demonstrating absence. They were used because time of death in wildlife is often unknown, and with time after death and autolysis, the probability of false negatives with these detection methods increases (18). However, these criteria may have led to an underestimation of the number of birds without trichomonads, but this would not exceed the number of inconclusive birds in this study and therefore still be a minority. Collectively, even though study design does not allow for inference on prevalence, these data showed that *T. gallinae* is commonly detected in greenfinches found dead in NL since 2009.

To assess the contribution of trichomonosis to the death of the greenfinches, the sample of NL birds was examined macroscopically and microscopically, distinguishing between lesions consistent with trichomonosis and lesions due to other causes. Trichomonads and associated lesions were only seen in the upper digestive tract, confirming the upper digestive tract as predilection site for trichomonosis in greenfinches, as described elsewhere (18). A fair proportion of the specimens with trichomonads in this Dutch convenience sample had no macroscopic lesions in the upper digestive tract, raising the question whether these were specimens with or without disease. Other studies have detected trichomonads by PCR in dead greenfinches in absence of macroscopic lesions, but without performing histology (28). Therefore, they could not distinguish between presence of disease or no disease (28). Here histology showed that the specimens with trichomonads all had microscopic ulcerative necrotic changes in the upper digestive tract, including those without macroscopic lesions. This provides evidence for disease. Further, there is no evidence for presence of the parasite in absence of disease. Therefore, histology is an essential part of the post-mortem examination process: all the greenfinches in the Dutch study in which trichomonads were detected can be considered to have the disease trichomonosis.

The comparison of the scores of the microscopic lesions at trichomonas predilection sites determined that ulceration was less deep and heterophil infiltration less extensive in the trichomonas-infected greenfinches with microscopic lesions only, compared to those with macroscopic lesions. The extent of macrophage infiltration and presence of bacteria was similar. These results collectively suggest a shorter, more acute disease course in the cases without macroscopic lesions, rather than less severe disease. Had there been less severe disease, one could also expect birds without macroscopic lesions to have other significant lesions due to other causes to explain their death. This was not the case. Rather, significant other lesions were equally rare in the trichomonad-infected groups with and without macroscopic lesions in the upper digestive tract. Therefore, there was no evidence that this difference in scores indicated less severe disease in greenfinches without macroscopic lesions. Fatal trichomonosis infections without macroscopic lesions (caseous changes) have been documented in other studies in birds of prey (29, 30).

Only a handful of greenfinches in this study was not infected with *T. gallinae*, and only a quarter of the birds with trichomonosis also had significant lesions due to other causes. The remainder only had trichomonosis, with hemorrhagic diathesis as a complication contributing to the deaths. Taking into consideration that not all studies include histology, trichomonosis was as frequently the cause of death in these Dutch greenfinches as in comparable published GB studies (23, 24). The hypothesis that in the Dutch sample trichomonosis would account significantly less frequently for greenfinch deaths than in greenfinches examined post-mortem in GB was rejected.

The second hypothesis in this study was that the partially typed trichomonas genomes would show genetic heterogeneity. The *T. gallinae* subtype A1 strain associated with finch trichomonosis in GB has been described as a very pathogenic strain (22). One possibility for less fatal disease in the NL, as in less impact of the disease at population level, is the presence of one or several strains of *T. gallinae* in Dutch greenfinches, that are different from the GB strain. Protective immunity to the virulent strain could possibly be acquired in birds infected early in life by less virulent strains (1, 4, 8–10). Assuming that strain diversity would be detected in greenfinches found dead, the study aimed to detect the presence of such a strain or strains by demonstrating genetic heterogeneity in sequences of the ITS1-5.8S-ITS2 gene region and the Fe-hydrogenase gene. The greenfinches infected with trichomonosis in GB and elsewhere in Europe have shown no genetic heterogeneity in the sequences of these two genes (22). The partial genotyping performed in this study showed the sequences in most of the strains cultured from Dutch greenfinches were identical to those of the GB strain. In three strains however there was a synonymous T-to-C substitution in the Fe-hydrogenase gene at position 135 compared to the “clonal” strain. This substitution is unlikely to be a reading error, as this study has used a high-fidelity DNA polymerase to limit reading errors, and the same substitution was found when the PCR test was repeated in each case.

The importance of this restricted genetic heterogeneity is unclear. A substitution that occurred during the culture process may be a possibility; however, it is not known if mutations occur during the culture process, but strains have been documented to lose their pathogenicity and infectivity while being cultured (1). Other studies have reported one or several single substitutions in the Fe-hydrogenase gene sequence compared to that of the GB finch strain, although not in greenfinches but in other bird species (7, 21, 30). One study hypothesized that this is due to multiple spill-over events among bird species (7). The three greenfinch cases in this study with the synonymous substitution were found in 2016 in different locations (Figure 2), and one of them belonged to an incident in which another and one of them belonged to an incident in which another greenfinch was typed as the clonal *T. gallinae* subtype A1 (Supplemental Material 2). To further elucidate the importance of this finding, other genes would need to be sequenced, preferably more variable than the SSU rRNA gene and the rpb1 gene, which are very conserved (21) and were therefore not used in this study. Primers have just been published for performing a parallel 19 locus multilocus sequence typing test that showed good discriminatory ability for distinguishing *T. gallinae* strains (31).

Trichomonosis cases were mostly reported during the spring and summer months; however, some cases did occur in autumn and winter. This is consistent with reports on finch trichomonosis in greenfinches in GB (24). Greenfinches infected with trichomonads were found widely across the NL. No inferences could be made on geographical spread over years, and this was never the focus of this study based on convenience sampling. It was not possible to perform robust fine-scale spatial analysis of trends and to identify spatial risk factors. This would require long-term garden bird health monitoring data, which may come within reach in the near future through a generally broad volunteer acceptance of the garden bird monitoring scheme in the NL. In addition, there were only a handful of the submitted dead greenfinches that were not *Trichomonas* cases. This lack of “negatives” also made it impossible to identify risk areas and risk factors.

Despite the numerous sources of bias in this study (convenience sample, depending on public for submission; applied case definitions; use of a culture step before PCR; and limited number of genes sequenced), the results imply that finch trichomonosis could be a threat to Dutch greenfinches that is temporarily masked by other factors in census data. Bird population dynamics are complex, and populations may show lagged responses to disturbance (32). There is still a poor understanding of the demographic, environmental, genetic and behavioral, factors that shape pathogen transmission and bird susceptibility (2). Bird density or garden bird feeding practices in the NL most likely differ from those in GB, affecting the transmission rate (24, 33). Creation of suitable habitat in urban areas could be boosting the Dutch greenfinch population in a positive direction. In addition, Dutch and British greenfinches may differ genetically from each other, affecting their immunity to disease. Alternatively, migrating greenfinches could temporarily be filling up niches emptied by the disease in the Dutch breeding population. For a better understanding of the threat and possible mitigation measures, it is relevant to investigate finch trichomonosis taking a multidisciplinary approach on a European scale.

## Data Availability Statement

The dataset generated for this study can be found in Supplemental material 2 and sequences were deposited in GenBank under the following accession numbers: MN385401, MN385402, MN385403, MN385404, and MN385405.

## Ethics Statement

Ethical review and approval was not required for the animal study because the animals were found dead and investigated in the context of a general wildlife disease surveillance programme.

## Author Contributions

JR, AL, MK, and AG: manuscript writing. JR: conception of study. AL, MK, and AG: pathology. AL: laboratory work. RS and JS: bird census data. JR and AL: data analysis. All authors critically reviewed the manuscript.

### Conflict of Interest

The authors declare that the research was conducted in the absence of any commercial or financial relationships that could be construed as a potential conflict of interest.

## Footnotes

^1^Dutch Wildlife Health Centre. https://www.dwhc.nl/groenling-het-geel/.
