# Supplementary material for: Trichomonosis in Greenfinches (Chloris chloris) in the Netherlands 2009–2017: A Concealed Threat
Source: Front Vet Sci. 2019 Nov 29;6:425. doi: 10.3389/fvets.2019.00425 (PMC6896826; doi:10.3389/fvets.2019.00425)

Supplementary Material 1

Supplementary Figure 1. Greenfinch population trends in the Netherlands. a: Breeding (resident) greenfinch population. b: Winter (migrating) greenfinch population. 1990 is chosen as reference year (index 100). Source: Dutch National Ecological Monitoring Scheme, Sovon Dutch Centre for Field Ornithology

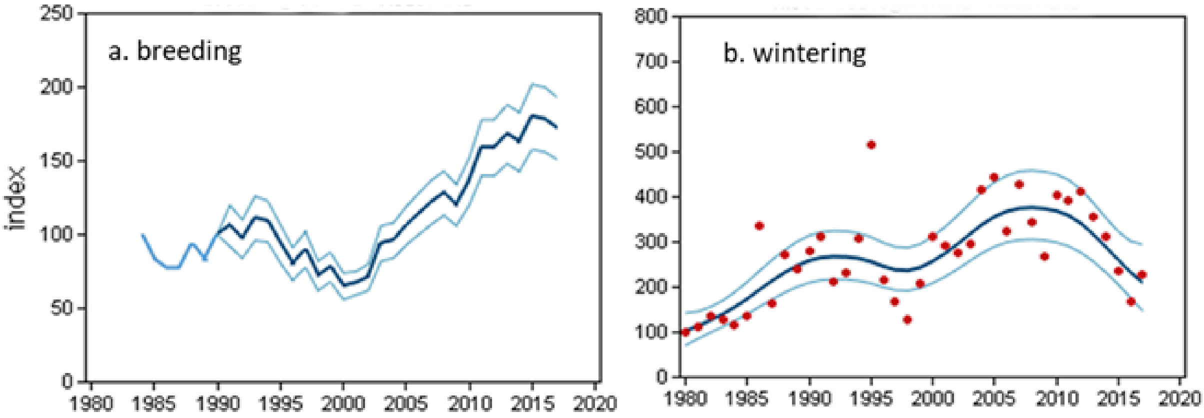

Supplement: Supplementary file 1 [file Table_1.pdf]
