# Supplementary material for: Trichomonosis in Greenfinches (Chloris chloris) in the Netherlands 2009–2017: A Concealed Threat
Source: Front Vet Sci. 2019 Nov 29;6:425. doi: 10.3389/fvets.2019.00425 (PMC6896826; doi:10.3389/fvets.2019.00425)
Supplement: Supplementary file 2 [file Table_2.pdf]

Supplementary Material 3

Table Supplementary Material 3. Tests performed per trichomonad status and concordance of tests.

Tricho-monad status	No. of tests performed	No. of birds	Cytology	Histology	Hanging drop	Concordance
Positive	3	14	+	+	+	Yes
		2	+	Inconclusive	+	NA
		4	+	-	+	No
		1	+	+	-	No
		3	-	+	+	No
		5	-	Inconclusive	+	NA
		1	Inconclusive	Inconclusive	+	NA
	2	21	ND	+	+	Yes
		2	ND	+	+ (later neg)	Yes
		5	ND	Inconclusive	+	NA
		1	ND	-	+	No
		1	ND	+	-	No
		2	+	+	ND	Yes
		1	+	ND	+	Yes
		1	+	ND	+ (later neg)	Yes
		1	+	ND	-	No
		1	Inconclusive	ND	+	NA
		1	-	+	ND	No
	1	5	ND	+	ND	NA
		21	ND	ND	+	NA
		2	ND	ND	+ (later neg)	NA
Negative	3	1	-	-	-	Yes
	2	4	ND	-	-	Yes
		1	-	ND	-	Yes
Inconclusive	3	2	-	Inconclusive	-	NA
	2	1	ND	Inconclusive	-	NA
	1	4	ND	ND	-	NA
		1	ND	-	ND	NA
		2	ND	Inconclusive	ND	NA
		1	-	ND	ND	NA
	0	11	ND	ND	ND	NA
Total		123				

Concordance between tests, after eliminating specimens with inconclusive test results:
No. of concordant tests / No. of concordant and not-concordant tests = 47/59 = 0.797 or 80%

Sensitivity cytology
Cytological results were available for 39 birds with a positive trichomonad status.
Of these 26 tested positive in cytology, the others tested negative (9) or inconclusive (2)
Se cytology = 26/37 = 0.70 or 70%

Sensitivity Histology
Conclusive histological results were available for 55 birds with a positive trichomonad status.
Of these 50 tested positive in histology, the others tested negative (5) or inconclusive (13)
Se histology = 50/68 = 0.74 or 74%

Sensitivity Hanging drop
Conclusive culture and hanging drop results were available for 87 birds with a positive trichomonad status. Of these 84 tested positive in the hanging drop, the other 3 tested negative.
Se hanging drop = 84/87 = 0.966 or 97%
