# Supplementary material for: Trichomonosis in Greenfinches (Chloris chloris) in the Netherlands 2009–2017: A Concealed Threat
Source: Front Vet Sci. 2019 Nov 29;6:425. doi: 10.3389/fvets.2019.00425 (PMC6896826; doi:10.3389/fvets.2019.00425)

Supplementary Material 4

Supplementary Figure 4. Seasonal distribution of 114 reported and 95 investigated greenfinch disease events with focus on trichomonosis

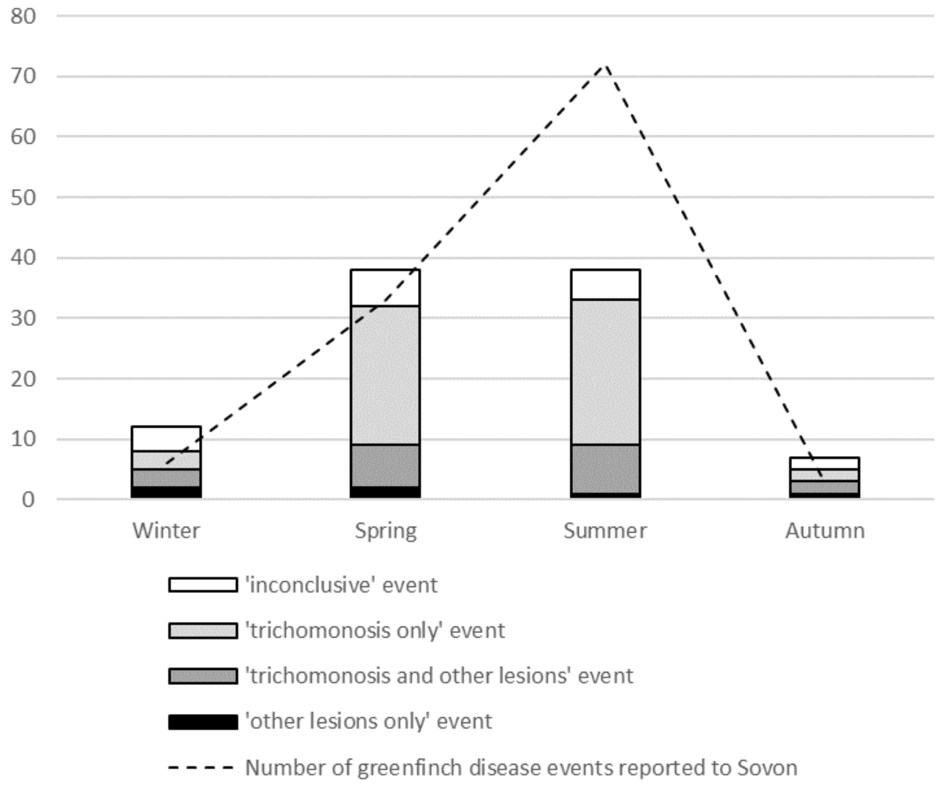

Supplement: Supplementary file 3 [file Table_3.pdf]
